# Borate Bioactive Glasses (BBG): Bone Regeneration, Wound Healing Applications, and Future Directions

**DOI:** 10.1021/acsabm.2c00384

**Published:** 2022-07-11

**Authors:** Duygu Ege, Kai Zheng, Aldo R. Boccaccini

**Affiliations:** † Institute of Biomedical Engineering, Bogazici University, Rasathane Street, Kandilli 34684, Istanbul, Turkey; ‡ Department of Materials Science and Engineering, Institute of Biomaterials, University of Erlangen-Nuremberg, 91058 Erlangen, Germany; § Jiangsu Province Engineering Research Center of Stomatological Translational Medicine, Nanjing Medical University, Nanjing 210029, China

**Keywords:** borate glasses, tissue engineering, wound healing, drug delivery, scaffolds

## Abstract

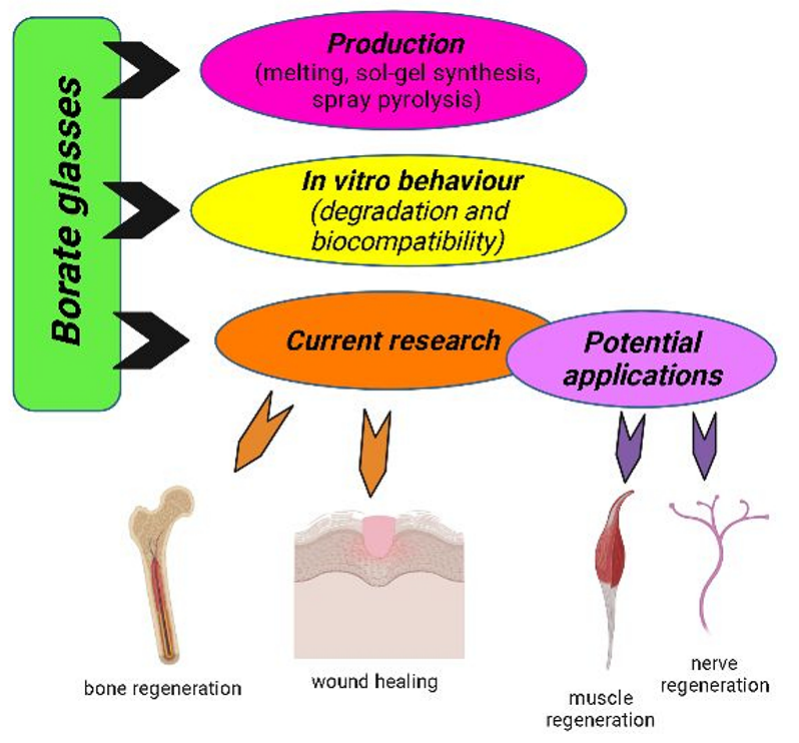

Since the early 2000s, borate bioactive glasses (BBGs) have been extensively investigated for biomedical applications. The research so far indicates that BBGs frequently exhibit superior bioactivity and bone healing capacity compared to silicate glasses. They are also suitable candidates as drug delivery devices for infection or disease treatment such as osteoporosis. Additionally, BBGs are also an excellent option for wound healing applications, which includes the availability of commercial (FDA approved) microfibrous BBG dressings to treat chronic wounds. By addition of modifying ions, the bone or wound healing capacity of BBGs can be enhanced. For instance, addition of copper ions into BBGs was shown to drastically increase blood vessel formation for wound healing applications. Moreover, addition of ions such as magnesium, strontium, and cobalt improves bone healing. Other recent research interest related to BBGs is focused on nerve and muscle regeneration applications, while cartilage regeneration is also suggested as a potential application field for BBGs. BBGs are commonly produced by melt-quenching; however, sol–gel processing of BBGs is emerging and appears to be a promising alternative. In this review paper, the physical and biological characteristics of BBGs are analyzed based on the available literature, the applications of BBGs are discussed, and future research directions are suggested.

## 1 Introduction

Bioactive glasses (BGs) are surface reactive materials when they are in contact with physiological fluids, such as human plasma, or in aqueous phosphate solution.^1−5^ In 1969, Prof. Larry Hench invented the first silicate-based BG, known as 45S5 BG (composition: 45SiO_2_–24.5CaO–24.5Na_2_O–6P_2_O_5_ in wt %).^6−8^ When soaked in human plasma (usually tested using simulated body fluid), an amorphous calcium phosphate (ACP) layer forms on the BG surface, which then crystallizes into hydroxyapatite (HA).^9^ This surface bioreactivity enables strong bonding with the surrounding bone tissue, which gives BGs their osteoconductive properties. Following the release of dissolution products, BGs are also osteoinductive.^10^ However, silicate-based BGs such as 45S5 and 13-93 (composition: 53SiO_2_–20CaO–6Na_2_O–12K_2_O–5MgO–4P_2_O_5_ wt %) glasses appear to have limitations for some applications. First, calcium phosphate (CaP) conversion is incomplete.^11^ In vivo, 45S5 BG transforms slowly to HA, and the conversion rate of 13-93 BG into HA is even slower. Another limitation is the likelihood of 45S5 BG to crystallize during heat treatments, which leads to difficulties producing noncrystalline 45S5-based 3D scaffolds and fibers.^12,13^

An important number of studies have shown that certain compositions of borate glasses (and phosphate glasses) are also bioactive.^9,14^ Borate bioactive glasses (BBGs) are produced by replacing network silica ions with boron ions in the glass network. Boron is an essential trace element with important roles in the human body.^15,16^ It is found in the body in the form of organoboron complexes of which 96% is boric acid and the rest is in the form of borate anion.^17^ It has been reported that 1 mg boron intake daily is optimum and essential for normal functioning of the body.^18^ In the body, bone, nails, and hair have the highest concentration of boron.^17^ Moreover, it has been reported that the presence of boron in the body alleviates symptoms of osteoporosis, coronary heart disease, and arthritis.^19,20^ Boron improves calcium integration into bone, joints, and cartilage.^21^ As part of the bone metabolism, boron works together with vitamin D, calcium, and magnesium, and it has anti-inflammatory and antioxidant effects.^22,23^ Moreover, boron has wound healing properties which are related to the ability of boron to regulate the release of collagen, proteoglycans, and proteins. Kerotinocyte migration is enhanced also in the presence of boron, which may play a key role in wound healing.^24^

**Figure 1:**
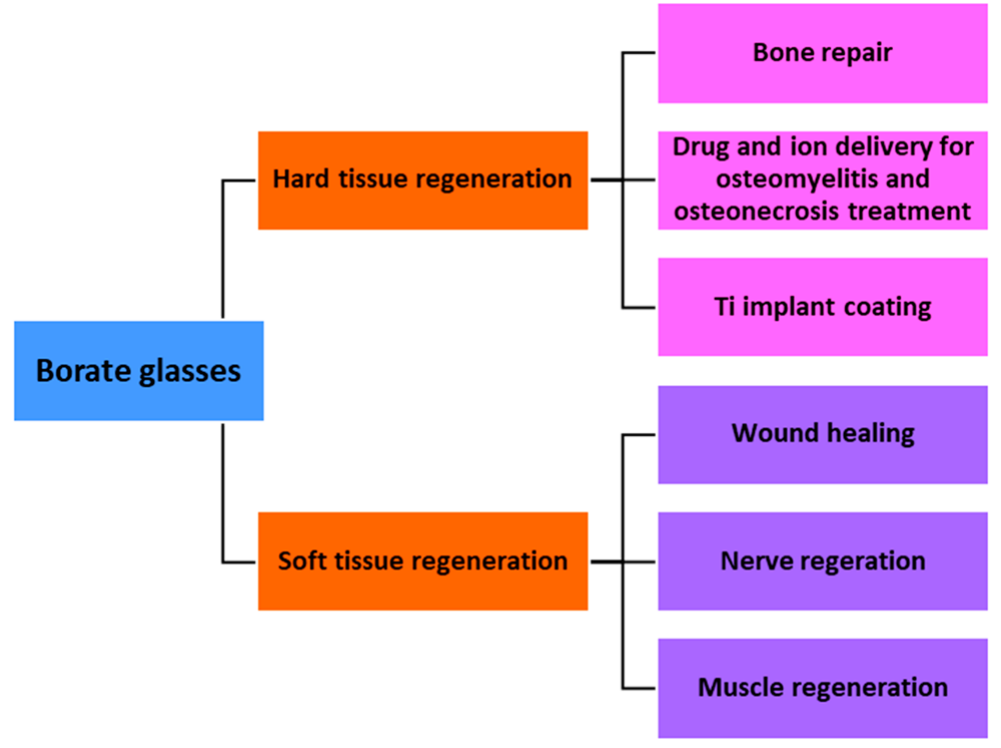
Applications of BBGs include soft tissue engineering (wound healing^29−31^ and nerve regeneration^32−34^) and hard tissue engineering applications.^35−37^

BBGs are the most recent members of the BG family.^25^ BBGs with specific compositions are biodegradable, bioactive, and osteoconductive.^26^ Due to their advantageous properties, in some cases surpassing the performance of silicate BGs, BBGs are exploited for bone regeneration, wound healing, and nerve tissue engineering applications.^27^ Such increasing interest in BBG applications in medicine prompted the preparation of this review. A search from 1990 to 2021 was performed with the search engines Web of Science, Google Scholar, and Scopus. BBGs were first developed for biomedical applications with a focus on bone tissue engineering at the beginning of the 2000s.^25^ Since then, BBGs have been increasingly investigated for a variety of biomedical applications, which is outlined in Figure 1.^28^ This review is organized in the following manner. First, the production of BBGs is introduced, which is followed by the discussion of the in vitro behavior of borate glasses. The next section reviews the effect of boron ion release from BBGs on cell viability. Then, the application of borate glasses in hard and soft tissue engineering is discussed. Finally, the scope for future research in the field is presented.

## 2 Processing Methods for BBGs

Generally, BGs are produced by the melt-quenching technique, which requires the melting of precursor oxide powders at elevated temperatures (above 1000 °C) followed by rapid cooling (quenching) of the melt to obtain an amorphous (noncrystalline) glass. BBGs can be produced as powders,^38−40^ which can be further processed to fabricate 3D scaffolds^41−43^ or microfibers.^30,44^ BBG scaffolds are usually produced by a polymer foam replication technique.^41,42,45^ Accordingly, microporous polyurethane scaffolds are immersed in a slurry of BBG powder dispersed in a solvent. The coated scaffold is then dried, and following this, the scaffold is heat-treated to remove the polymeric phase and sinter the BBG struts.^41,42,45^ Cotton-like microfibers based on BBGs have also been produced by exploitation of the melting technique.^30,44,46^ This type of BBG microfiber has been FDA-approved and commercialized with the trade name Mirragen for wound healing applications.^10,47,48^

**Figure 2:**
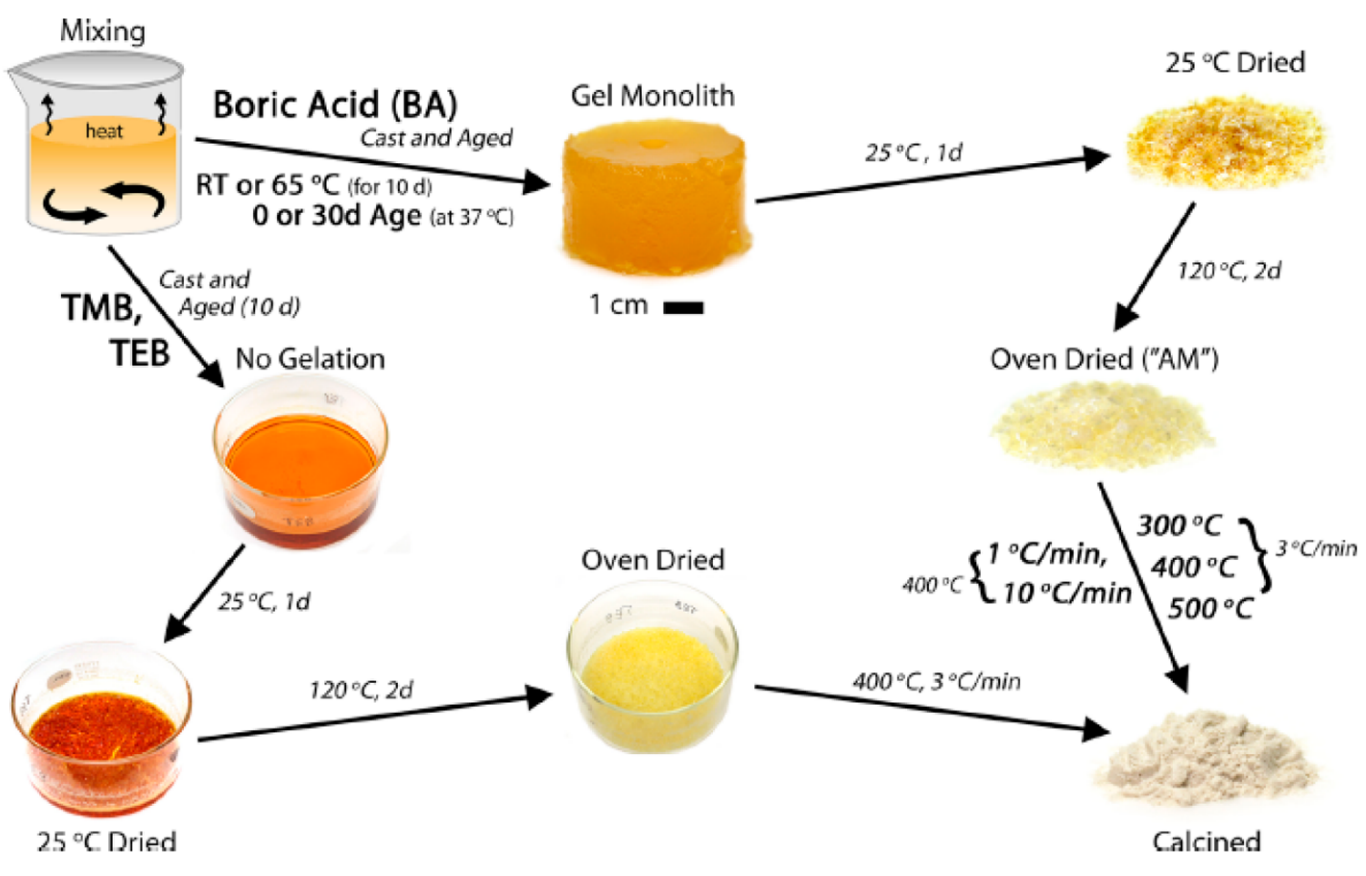
Schematic diagram showing the sol–gel processing of BBG^58^ (Reproduced with permission from ref (58). Copyright 2015 American Chemical Society).

The most commonly studied BBG obtained by the melt-quenching technique for biomedical applications is the 13-93B3 composition (54B_2_O_3_–22CaO–8K_2_O–8MgO–6Na_2_O–2% P_2_O_5_ in mol %).^9,29,49−54^ This glass was developed with the base composition being the silicate 13-93 BG and replacing silica with borate ions. During melting, control of the composition of the glass is challenging because of the presence of volatile components. Also, in general, contamination may take place during melting and crushing. Moreover, control of the morphology and mean particle size of melt-derived BGs is challenging. As a result, the sol–gel process has also been considered for the preparation of BGs.^55^ The sol–gel route exploits liquid-based precursors to enable gelation of the glass network via hydrolysis and condensation reactions. Subsequently, the gel is dried and calcined to densify the amorphous glass and remove any organic product.^13,56,57^ Lower network connectivity (NC) makes gelation of BBGs difficult. Only a few studies are available reporting on the sol–gel processing of BBGs. In fact, boron had been previously exploited only as a network modifier. In 2015, the first sol–gel precipitated BBGs were produced with composition 46.1B_2_O_3_–26.9CaO–24.4Na_2_O–2.6P_2_O_5_ in mol %.^58^ In comparison with melt-quenching, sol–gel processing leads to the production of at least 2 orders of magnitude greater specific surface area and total pore volume of BGs, which dramatically increase the extent of aqueous interactions and ion release rates. Other advantages of sol–gel derived BGs include improved purity, homogeneity, and reduced processing temperatures.^58^ Moreover, sol–gel processing leads to production of BBGs with a rough, nanoporous texture which is in contrast to the smooth surface appearance of melt-derived glasses.^56^Figure 2 schematically shows the sol–gel processing route for BBGs introduced by Lepry et al.^58^

The effects of different ions have been analyzed for sol–gel processed BBGs. Network modifiers such as sodium and potassium disorganize the glass matrix, and a high sodium content leads to glass crystallization at reduced temperatures.^59^ Additionally, low borate containing glasses undergo earlier crystallization due to the greater extent of densification at lower temperatures.^58^ On the other hand, higher borate content glasses remain amorphous at higher calcination temperatures which implies that high borate contents favor glass formation. Lower borate content glasses exhibit fewer boron units, which leads to more terminal groups, specifically OH^–^. These terminal groups are more susceptible to interactions with the phosphate solutions resulting in their faster degradation in comparison to higher borate content glasses. A faster degradation is more pronounced for sol–gel processed glasses, as terminal groups are not completely eliminated during drying and calcination.^58^

Recently, Deliormanlı et al.^60^ fabricated 13-93B glasses by the sol–gel method. Lepry et al.^61^ had also prepared binary glasses in the CaO–B_2_O_3_ system by the sol–gel route previously. All of the glasses prepared had high surface area and exhibited nanoporosity.^61^ Another method to produce BBG is the use of high temperature spray pyrolysis by which particles can be achieved of size smaller than 1 μm.^41^ In this method, ultrasonic spray generators are used to atomize the precursor solution which is introduced into a hot reaction column where droplets are dried, decomposed, and crystallized.^55,62^ Cho et al.^55^ successfully produced 45S5B1 BBG (46.1B_2_O_3_–24.4Na_2_O–26.9CaO–2.6P_2_O_5_ in mol %) particles by using high temperature spray pyrolysis, thus obtaining nanometric particles with high surface area.

**Figure 3:**
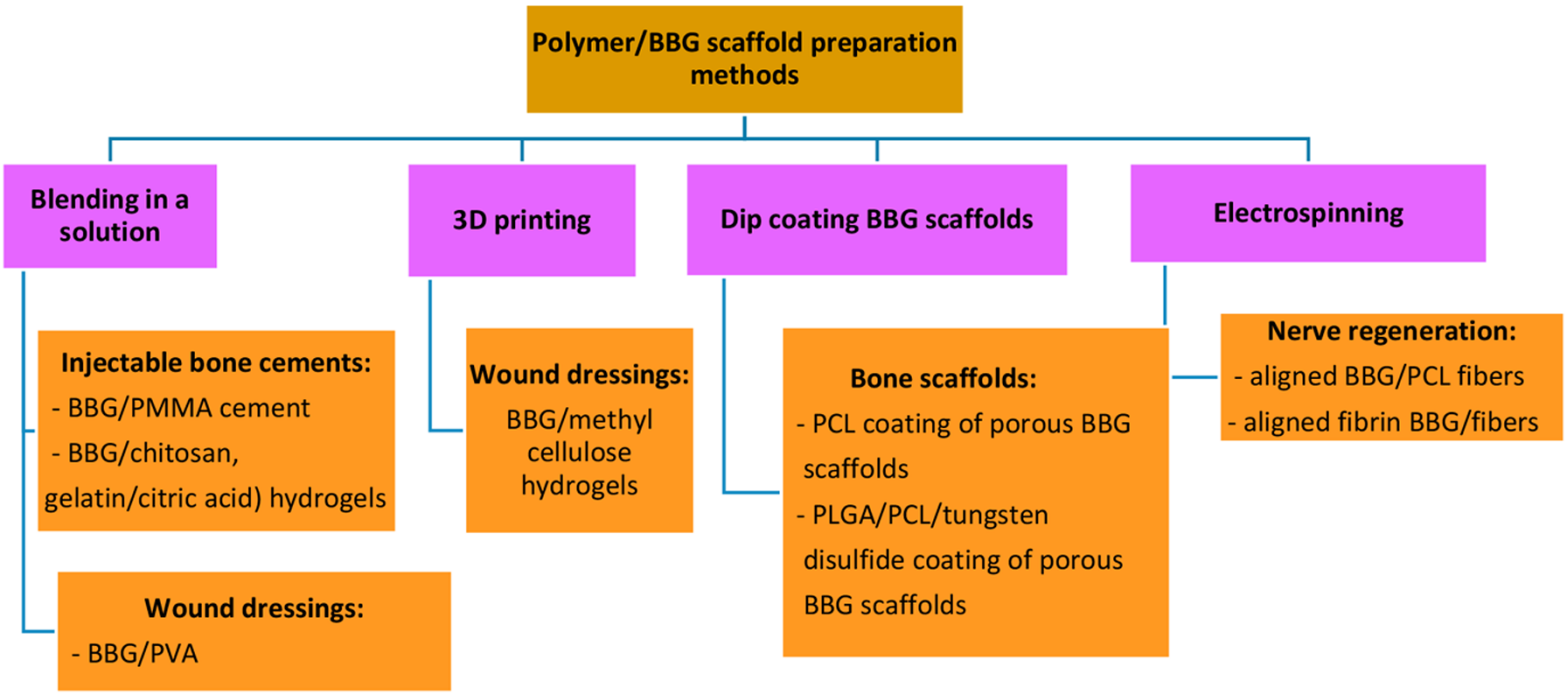
Production methods for preparation of BBG-polymeric scaffolds for various applications.^10,26,63−65,69−71^

BBGs are also used together with polymers, forming composites, for various applications. For preparation of bone cements, initially BBGs were mixed with PMMA powder, which was then combined with the liquid component for polymerization and subsequently pressed into a mold to form a block.^10^ BBGs have also been blended with chitosan solution to form an injectable scaffold to heal bone defects.^26^ For similar applications, BBG incorporated gelatin-based injectable scaffolds were prepared by mixing gelatin and citric acid with BBG powder.^63^ To increase the mechanical properties of porous BBG scaffolds, they were coated with PCL in a solution of PCL–acetone for 30 min.^64^ Similarly, in another study BBG scaffolds were coated with tungsten disulfide/PLGA/PCL by the dip coating method.^65^ For wound healing applications, BBG/PVA hydrogels have been prepared by blending in solutions.^66,67^ BBG/methyl cellulose/manuka honey hydrogels were also 3D printed for wound healing applications.^67^ For nerve regeneration applications, BBG powders were mixed in a PCL solution which was electrospun into aligned fibers.^68^ The main types of production methods for preparation of BBG/polymeric scaffolds are summarized in Figure 3.

## 3 Properties of Borate Bioactive Glasses

### 3.1 Acellular Bioactivity

The bioactivity of BGs is usually evaluated by their conversion rate to HA when the materials are immersed in simulated body fluid (SBF) for periods which may vary from hours to months, depending on the composition of the glass.^14,72^ The conversion of BBG to HA occurs via dissolution–precipitation reactions similar to the ones occurring in silicate glasses, but without the buildup of a silica-rich layer.^11,36,73^ The concept of bioactivity is relevant for applications in contact with bone tissue as the formation of HA is the marker that characterizes strong bonding of a material to bone. Initially, the glass converts to HA via a surface reaction. The degradation and conversion of BBG to HA in SBF occur by dissolution of ions into the solution and the reaction of calcium ions from the glass with phosphate ions from the solution to form ACP and then a crystalline HA layer on the glass surface.^26^

The continuous dissolution–precipitation reaction results in the growth of the HA layer gradually inward from the surface.^1,11,53^ This reduces the volume until complete conversion of the BBG to HA.^58^ This process is controlled by diffusion of calcium and phosphate ions to the reaction interface or reaction of calcium and phosphate ions at the interface.^13^

**Figure 4:**
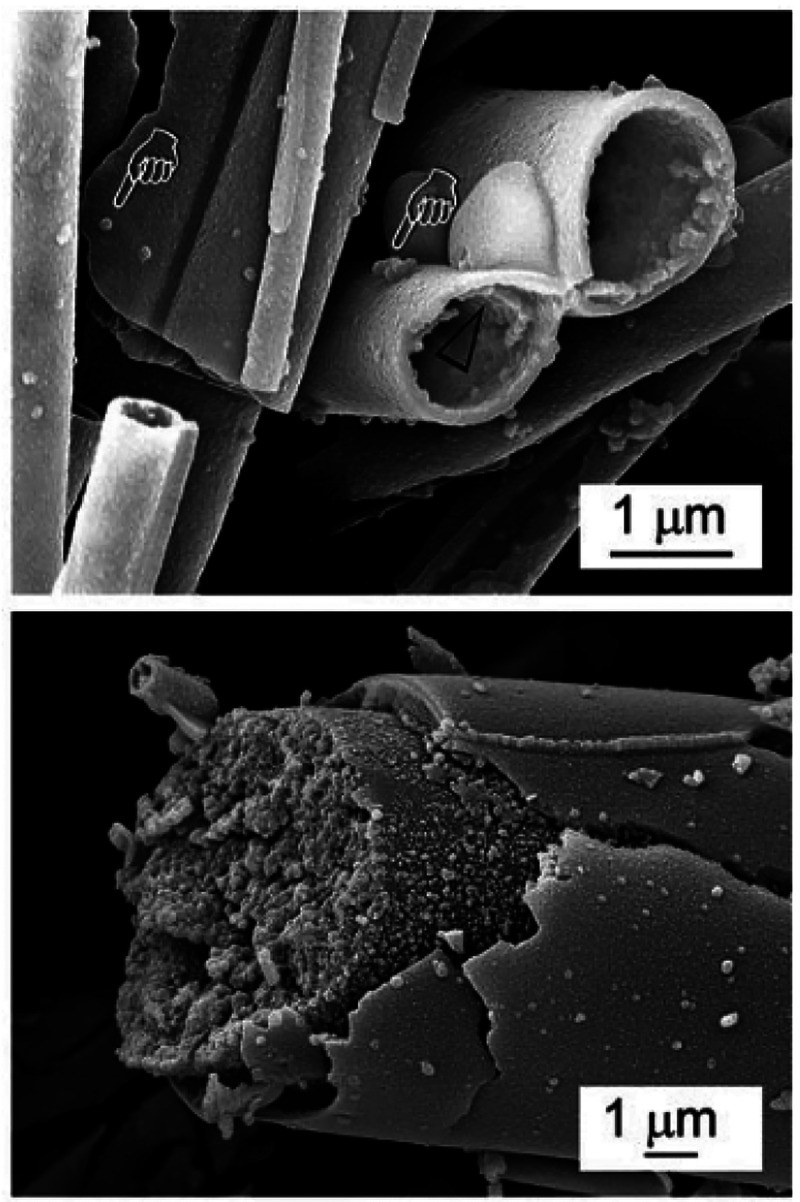
SEM images of microfibrous BBG (arrows indicate extrafibrillar calcium phosphate globules) before and after immersion in SBF for 4 days^75^ (Reproduced with permissions from ref (75). Copyright 2015 Royal Society of Chemistry).

The mixture of trigonal planar [BO_3_] and tetrahedral [BO_4_] units in BBGs is less durable than tetrahedral SiO_2_ units in silicate glasses due to reduction of network connectivity.^10,58,69,74^ Therefore, 13-93B3 BG, for example, degrades more quickly than silicate glasses and converts more completely into HA.^10,26,52,72,74^ Liang et al.^36^ observed a white layer formation on their BBGs only after 10 min of immersion in SBF, and after 7 days, complete conversion to HA had been achieved. SEM and XRD analyses usually demonstrate a visible HA layer after 24 h in SBF for BBG. In comparison, for silicate glasses, the HA layer was still not visible after 7 days in SBF.^57^ Glasses based on the B_2_O_3_–CaO–Na_2_O–P_2_O_5_ system with a wide compositional range (36–61 mol % B_2_O_3_) were reported to rapidly convert to bone-like mineral (CaP) in SBF.^56^Figure 4 shows unconverted microfibrous borate glass (BG) (53.8 B_2_O_3_, 20.0CaO, 12.1 K_2_O, 4.6 Na_2_O, 4.6 MgO, 3.8 P_2_O_5_ in wt %) and partially converted microfibrous borate glass after immersion in SBF for 4 days.^75^

### 3.2 Degradation Behavior of BBGs

**Figure 5:**
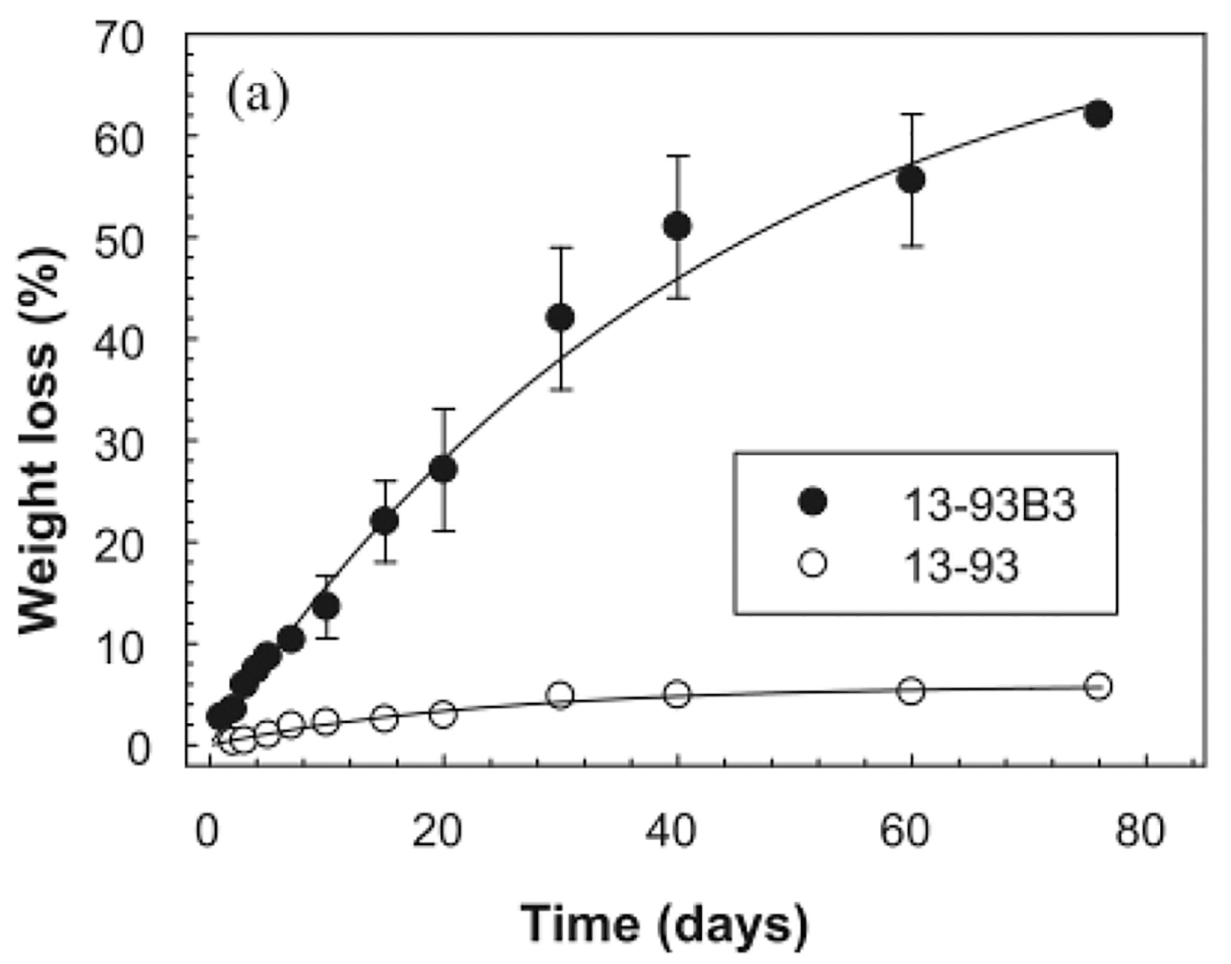
% Weight loss of silicate 13-93 and borate 13-93B3 scaffolds in SBF^52^ (Reproduced with permissions from ref (52). Copyright 2012 Elsevier).

A scaffold for tissue engineering has to sustain its structural integrity and mechanical strength until tissue formation has occurred. Therefore, controlling the degradation behavior of scaffolds is critical in tissue engineering applications.^53^ Pramanik et al.^75^ indicated that for BBGs, the % weight loss of the scaffold was most rapid in the first day and increased with SBF immersion time. Another study illustrated that after 1 week in SBF, more than 90% of the glass degraded to form poorly crystallized HA.^76^ Additionally, the % weight loss for 13-93B3 scaffolds was drastically higher than for the silicate 13-93 and 45S5 scaffolds.^36,52^Figure 5 shows the difference of % weight loss of 13-93 and 13-93B3 BG scaffolds.^52^

**Figure 6:**
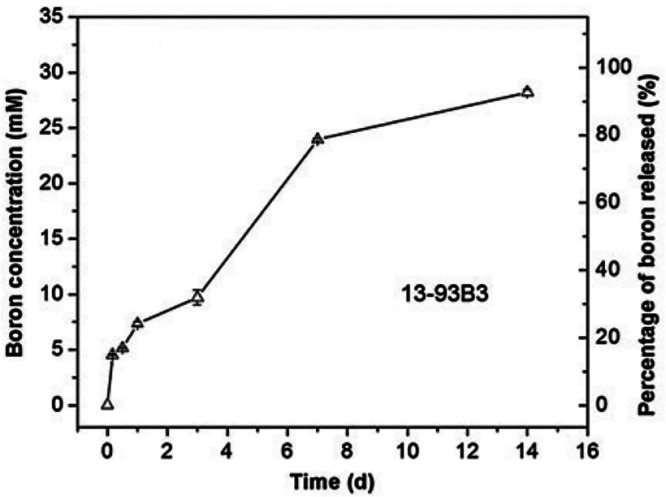
Concentration of boron ion released from 13-93B3 fibers into SBF at 37 °C as a function of time^9^ (Reproduced with permissions from ref (9). Copyright 2013 Springer).

According to the study of Liu et al., after 1 day in SBF, approximately 35% of boron ions of the scaffold were released. After 7 days, approximately 80 wt % of boron was released which reached 90 wt % release after a week.^9^Figure 6 shows the time-dependent concentration of boron ion release from 13-93B3 microfibers in SBF.^9^

Gu et al.^49^ found that increasing the B_2_O_3_ content increased the degradation rate, but the capacity of the scaffolds to support the proliferation of osteogenic cells during conventional culture in vitro decreased. After 3 days in SBF, a higher concentration of calcium ions was released from 13-93B3 than from 45S5 BG microfibers. Within 7–14 days, 13-93B3 microfibers degraded almost fully and converted to ACP, whereas only 15% degradation occurred in the 45S5 BG microfibers. After this, ACP on 13-93B3 microfibers crystallized more slowly to HA than the ACP on 45S5 BG microfibers.^9^ Studies also indicated that the scaffolds with partial conversion to HA were more favorable for cell viability. Therefore, the relatively slow crystallization feature of BBGs may be perceived as an advantage for improved biocompatibility.^36^

For silicate glasses, the addition of modifier oxides always changes bridging oxygen atoms to nonbridging oxygen atoms which reduces network connectivity. In the case of BBGs, first, the interconnectivity rises with the addition of modifier cations due to their interaction with negatively charged BO_4_ tetrahedra. If more modifier cations are added to the BBG, the BO_4_ groups change back to BO_3_ groups, and therefore the number of nonbridging oxygen ions increases and the network connectivity is reduced. This is called the borate anomaly in the literature.^29,61^ A high amount of modifiers with lower network connectivity reduces chemical durability and increases the dissolution rate.^29^

The incorporation of different ions in BGs is important to alter the BG degradation behavior and bioactivity. For example, the substitution of calcium ions by magnesium ions distorts the matrix structure, as magnesium is a smaller ion than calcium. Even small concentrations of magnesium ions can increase the stability of ACP. Therefore, poorly crystallized HA was reported to form on 13-93B3 scaffolds due to magnesium ion incorporation.^9,69^ Although magnesium ions decrease HA’s crystallinity, this effect could support bone growth and attachment considering that magnesium ions encourage osteoblast formation, differentiation, and adhesion, thus supporting bone regrowth. Magnesium ions should also improve the attachment of bone to the biomaterial’s surface.^19,44^

When BBGs are immersed in a phosphate solution, the pH increases abruptly with time and eventually reaches a plateau which may favor the in vitro formation of HA. The change of pH value from 7 to 10 indicates the ion exchange between the hydrogen in phosphate buffer solution and the BG surface.^66^ Sodium and calcium ions exchange with hydrogen ions at the initial dissolution stage, which leads to a pH increase.^29,52,75^ For BBGs, a higher % weight loss of the glass sample leads to a higher pH of the phosphate solution as a function of time.^11^ The studies indicate that the pH of the solution increases more rapidly when the B_2_O_3_ content of the glass increases.^72^

**Figure 7:**
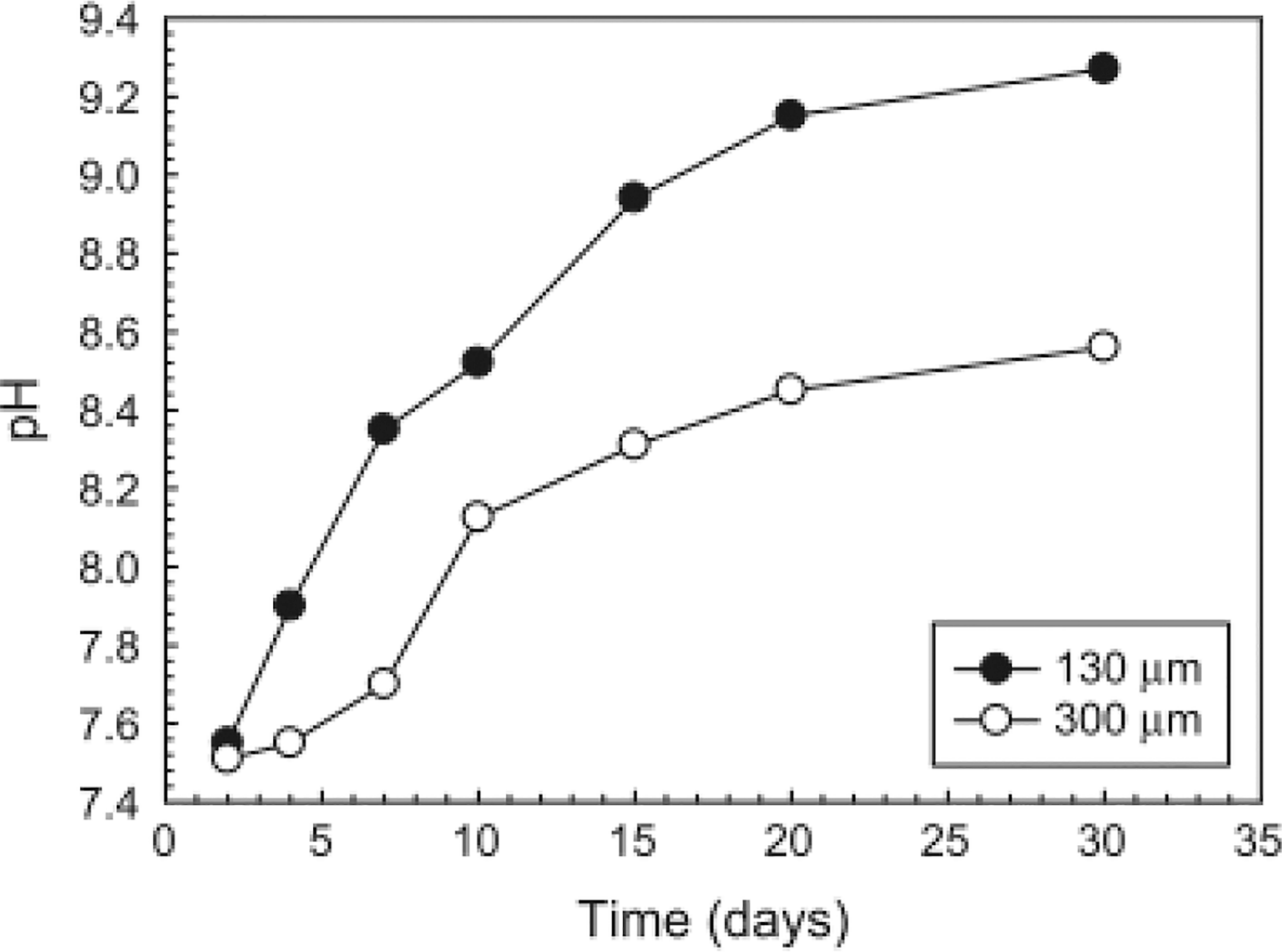
pH of SBF solution for 13-93B3 particles with two different particle sizes^53^ (Reproduced with permissions from ref (53). Copyright 2013 Springer).

Deliormanli et al.^53^ prepared BBG scaffolds with different strut sizes by 3D printing. As shown in Figure 7, after soaking of BBG scaffolds in SBF for 30 days, a pH increase up to 9.26 and 8.56 with strut diameters of ∼130 μm and ∼300 μm, respectively, was observed. This shows that the strut size of BBG scaffolds has a strong influence on ion dissolution rates. Smaller particles form more apatite and degrade more completely than larger particles. Another study performed by Zhang et al.^78^ showed that the particle size had a strong influence on the pH changes during BG degradation in SBF. During 3D printing, larger particles showed a smaller increase in pH but clearer reaction layers than smaller particles.^53^ In in vivo conditions, the ions would probably diffuse farther, which may lessen ionic concentrations and increase BBG’s rate of degradation, ultimately diminishing relatively high pH changes.^10^

### 3.3 Cell Biology Characterization of BBGs

**Figure 8:**
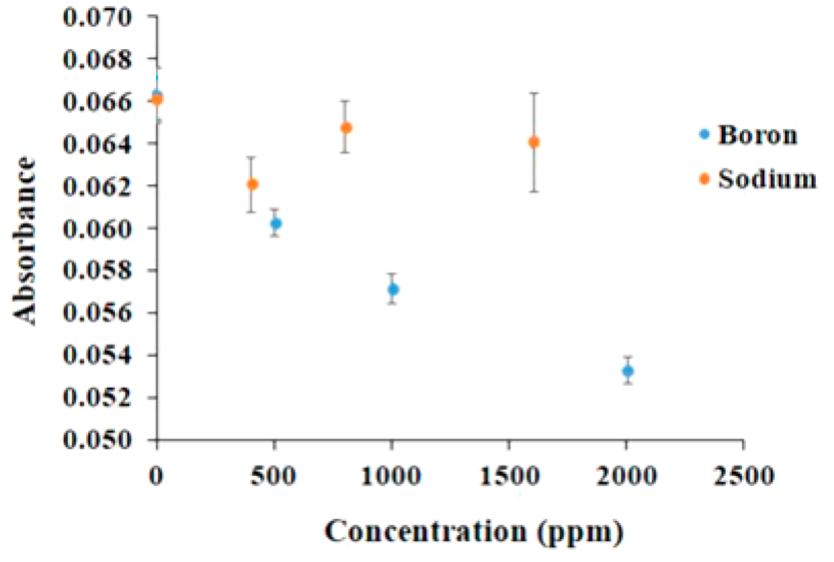
Absorbance values of preosteoblastic MC3T3-E1 cells cultured on different concentrations of boron and sodium ions^41^ (Reproduced with permissions from ref (41). Copyright 2021 Wiley).

The concentration of ions released from BBGs may have a significant impact on cell proliferation.^41^ Boron is present in the daily diet constituting an essential trace element in the human body. Trace quantities of boron are required for optimal health. Furthermore, boron dissolves rapidly in the body fluid and can be excreted in the urine.^13^ Boron has a positive influence on embryogenesis, immune function, and psychomotor skills.^12^ Moreover, the controlled release of boron during degradation of BBGs can improve bone repair, since small concentrations of boron are reported to favor bone growth.^9^ However, some studies show that trace metallic elements are potentially cytotoxic.^51,79^ High concentrations of boron can have a significant negative impact on the brain and reproductive health.^66^ One of the concerns related to the medical use of BBGs is the release of borate ions during degradation of the glass.^80^ Studies indicate that increase of the amount of borate ions in glass reduces cell density.^81^ Brown et al.^82^ showed that above a threshold concentration of ∼16 mM, borate ions leaching out of glasses (56.1B_2_O_3_–26.9CaO–24.4Na_2_O–2.1P_2_O_5_ mol %) inhibited the proliferation of MC3T3-E1 cells. Even a concentration of 2 mM boron ions led to a reduction of 40% of the cell density. Parallel with this, in another study, BBG scaffolds (52%B_2_O_3_–12%CaO–6%P_2_O_5_–14%Na_2_O–16%ZnO–*x*TiO_2_) were incorporated with 5, 15, and 20 mol % of titanium oxide ions and the % viability of MC3T3-E1 cells was evaluated. Cell culture studies indicated that the % cell viability decreased after treatment with BBG scaffolds over a 30 day period. To be able to observe the source of reduction of % cell viability, MTT tests were conducted with various concentrations of sodium and boron ions, separately. MTT tests revealed that the % cell viability decreased gradually with the increase of released boron concentration from 500 to 2000 ppm, while sodium ions showed no such toxicity. Figure 8 shows % cell viability after incubating cells in different concentrations of boron and sodium ions.^41^

In a few other studies, boron ion release from borosilicate glasses was investigated. These studies are also beneficial to determine the effect of boron ions on cell proliferation.^72,83^ Fu et al.^72^ studied the effect of boron concentration released from 13-93B2 BG (22CaO–6Na_2_O–8MgO–8K_2_O–18SiO_2_,36B_2_O_3_–2P_2_O_5_) extracts on bone marrow mesenchymal stem cell (BMSC) and MLO-A5 cell viability. The tested boron concentrations were 0.650, 1.301, 2.601, and 5.204 mM. In agreement with the other studies, a gradual decrease of % cell viability with the increase of boron concentration was observed. While the highest boron concentration of 5.204 mM was found to be toxic, 0.65 mM boron concentration was nontoxic for the seeded cells. Finally, Liu et al.^83^ indicated that release of boron ions from 13-93B2 glass with a concentration lower than 105.1 ppm was nontoxic, and it induced proliferation of BMSC. When the concentration units are converted, the results of all authors are observed to be in the same range and support each other, indicating that there is a maximal boron concentration that can be beneficial (in vitro).

Overall, all available data indicates that it is critical to design BBG scaffolds with a suitable boron ion release rate to induce proliferation of cells. The toxic effect on cells could be reduced by partial conversion of the BBG to HA prior to cell culture or the use of more dynamic cell culture conditions. For reduction of cytotoxicity, Chen et al.^84^ coated borate glass (53 wt % B_2_O_3_, 20 wt % CaO, 6 wt % Na_2_O, 5 wt % MgO, 12 wt % K_2_O, and 4 wt % SrO) with hydroxycarbonate apatite by immersing borate glass in a buffer solution 4.2 mM NaHCO_3_, 1 mM KH_2_PO_4_/K_2_HPO_4_, and 2.5 mM CaCl_2_ under dynamic conditions. Although some boron concentrations show toxicity in the mentioned static in vitro conditions, the same concentrations have very good performance in dynamic conditions, which was proven with many in vivo studies conducted, and these are discussed in the following section.^82^ The reason for this is the dilution of local boron concentrations in a dynamic environment.

## 4 Applications of BBGs

The two main areas of research in which BBGs are being considered are bone repair and wound healing.^85^ As early research indicated that BBGs were highly bioactive, bone regeneration applications were started to be investigated in the early 2000s.^25,28^ After a decade, Jung et al.^86^ observed wound healing capability of BBGs. After this, BBGs were heavily investigated for skin regeneration. Some other potential applications in soft tissue engineering have emerged such as nerve,^33,68^ muscle,^86^ and cartilage regeneration.^82^

### Hard Tissue Applications (Bone Regeneration)

Many studies have reported that BBGs could contribute to regenerate bone with no cytoxicity in vivo.^51,58,79,80^ The controlled release of boron during degradation of BBGs can improve bone repair since small concentrations of boron favor osteogenesis.^9^ The ion release and degradation rates of 13-93B scaffolds have been reported to trigger bone formation and resorption.^9^ Calcium and other ions released during BBG conversion activate osteogenic gene expression. Interestingly, BBGs simulate angiogenesis which sustains transportation of precursor cells, oxygen, growth factors and essential nutrients, and, thus, the growth and maintenance of new bone can be established.^11,19,55^

As mentioned above, the buildup of an HA layer on biomaterials in vitro suggests the bioactive potential of BBGs in vivo.^11^ Shorter conversion times in vitro could indicate more rapid healing which has been illustrated in bone defect models, in vivo.^1,56,61^ Radiographic images of Xie et al.^80^ showed that 13-93B3 scaffolds were mostly reabsorbed and replaced by a large amount of new bone while calcium sulfate was completely reabsorbed and replaced by a modest amount of new bone. BBG scaffolds with controlled and complete degradation behavior were biocompatible and had higher bioactivity in comparison to silicate-based BGs. BBGs also supported the growth and differentiation of human mesenchymal stem cells enhancing their suitability for bone tissue engineering.^80^ In another study, microfibrous silicate 13-93 and borate 13-93B3 scaffolds were implanted in rat calvarial defects. After 12 weeks, it was shown that while 13-93 fibers were only partially converted to HA, 13-93B3 fibers were fully converted.^49^

It has also been reported that BBGs form strong bonds with titanium.^37,88,89^ BBGs also have a potential preventive effect on bisphosphonate-related osteonecrosis of the jaw, receiving increasing research interest. Real-time quantitative polymerase chain reaction studies have indicated that zoledronic acid and BBG (53.8 B_2_O_3_–20.0 CaO–12.1 K_2_O–4.6 Na_2_O–4.6 MgO–3.8 P_2_O_5_ in wt %, GL1550) led to increase osteogenic and angiogenic gene expressions of BMSC and human endothelial (HUVEC) cells, respectively, compared to the control group (with no zoledronic acid or BBG treatment).^77^

The compositional flexibility of BBGs enables the possibility to add biologically active ions to its structure.^70^ For instance, calcium and silicon ions stimulate osteoblast differentiation.^4,14^ Strontium is known to favor bone growth.^70,90^ Zhang et al.^90^ produced 9 mol % strontium ion incorporated 13-93B3 bone cements. Strontium incorporated BBG was observed to improve the osteogenic differentiation of human bone marrow derived mesenchymal stem cells in vitro compared with pristine 13-93B3 incorporated bone cements. Copper exhibits angiogenic properties.^43^ Rahaman et al.^73^ produced scaffolds using 4% copper oxide incorporated 13-93B3 BG. These scaffolds were incorporated into a rat calvarial defect model. Copper incorporated scaffolds led to higher bone growth compared with pure 13-93B3 scaffolds. A higher amount of bone formation for copper incorporated scaffolds was attributed to their angiogenic properties provided by the presence of copper ions. Graphene platelets have been also incorporated into BBG scaffolds. The results indicated that addition of 5% graphene led to the optimum in vitro response with induction of electrical conductivity which was measured as 0.06 S/cm. Authors stated that such electrically conductive scaffolds are promising candidates for bone tissue engineering applications.^1^Table 1 shows a summary of BBG based systems reported in the literature incorporating different dopants and intended for bone tissue engineering applications.

**Table 1:** Incorporation of Different Ions in Melt Derived BBG Scaffolds for Bone Healing

composition	dopant	cell culture studies	other findings
BBG scaffold (40B_2_O_3_–[20 – *x*]CaO–25Li_2_O–15P_2_O_5_–*x*MgO)	0.5, 1, 2, 3 mol % magnesium ions	MG-63 cell viability did not decrease with increasing concentrations of magnesium ions	Magnesium ions increased degradation rate of BBG. This improved hardness and wear resistance. A hardness of 5.79 MPa was achieved^91^
(60B_2_O_3_–[40 – *x*]CaO–*x*MgO)	5, 10, 20, 40 mol % magnesium ions	MC3T3-E1 cell viability decreased above 20% magnesium doping	High bioactivity was achieved up to 10% magnesium. Specific surface area and pore volume reduced above 20 mol % magnesium incorporation^92^
BBG scaffold (52B_2_O_3_–12CaO–6P_2_O_5_–14Na_2_O–16ZnO–*x*TiO_2_)	5, 15, and 20 mol % of titanium oxide ions	MC3T3-E1 cell viability decreased with concentration of boron up to 2000 ppm.	Titanium oxide incorporation controls degradation and ion release rates which was stated to be advantageous for bone tissue engineering applications.
			Zinc ions induced antibacterial effect^41^
BBG scaffold (52B_2_O_3_-12CaO–6P_2_O_5_–14Na_2_O–16ZnO–*x*TiO_2_)	5, 15, and 20 mol % of titanium oxide ions	N/A	Porous structures were achieved by polymer foam replication method. Titanium oxide incorporation was found to be effective to control degradation rate and mechanical properties.
			Compressive strength: 9 MPa^42^
BBG coating (59B_2_O_3_–13P_2_O_5_–3CaCO_3_–15Na_2_CO_3_–10TiO_2_–SrCO_3_)	0, 15, 25 mol % strontium ions	N/A	Strontium ions significantly improved fracture toughness of BBG coatings^93^
13-93B3 frits	0.75, 1, 2 wt % silver oxide ions	MC3T3-E1 cell viability decreased with addition of 2 wt % silver ions.	Silver ion doped BBG had antibacterial effects^94^
BBG powder (59.5B_2_O_3_–2P_2_O_5_–9.5CaO–9CaF_2_–(20 – *x*)Na_2_O)–*x*Ag_2_O	0.25, 0.5, 0.75 mol % silver oxide ions	MC3T3-E1 cell viability decreased only for the highest silver ion concentration of 0.74 mol %	Silver ion doped BBG had antibacterial effects. Silver oxide had no influence on bioactivity^38^
BBG powder (45B_2_O_3_–24.5CaO–24.5Na_2_O–6P_2_O_5_) with metal oxide dopant	2 mol % zinc oxide, titanium oxide, tellurium oxide or cerium oxide	Low toxicity was measured with fibroblast cells	Tellerium doped BBG had the highest antibacterial activity against methicillin-resistant *Staphylococcus aureus*(95)
BBG scaffold (52.6B_2_O_3_–6Na_2_O–12K_2_O MgO–5CaO–20P_2_O_5_–0.4CuO)	4 mol % copper oxide ions	N/A	Trabecular, fibrous, and oriented structures were produced. Fibrous scaffolds led to higher bone formation than other structures in vivo^73^
Porous BBG scaffold (13-93B3 doped with copper oxide)	0.5, 1, and 2 mol % copper oxide ions	Scaffolds were biocompatible up to 1% copper oxide doping	Upto 2% copper oxide incorporation increased compressive and flexural strength and toughness^96^
(54.90B_2_O_3_–17.95CaO–5.34Na_2_O–10.77K_2_O–4.46MgO–3.59P_2_O_5_–3V_2_O_3_) scaffold	3 mol % vanadium oxide ions	N/A	Vanadium ions increased degradation rate and bioactivity but reduced mechanical properties^97^
13-93B3 powder doped with gallium oxide and zinc oxide ions	1, 3, and 6 mol % zinc oxide and gallium oxide	Concentration-dependent cytotoxicity with MG-63 osteoblast cells	Zinc ion doped BBG had higher antibacterial activity against *S. aureus*(98)

Osteomyelitis, which is the serious bacterial infection of bone, may occur at any age; however, diabetic patients are found to be particularly susceptible.^87^ This infectious disease is very difficult to cure, and the treatment for osteomyelitis includes removal of the infected area of the bone followed by a long duration of intravenous antibiotic treatment. However, intravenous delivery may be inefficient to reach avascular areas in the infected bone. Therefore, local delivery of high doses of antibiotics may be preferable in the treatment of osteomyelitis. Antibiotic-loaded calcium sulfate is commercially available for clinical use in the treatment of osteomyelitis. Calcium sulfate has shown to be predictable and high release rates of antibiotics due to its high degradation rate; however, it is found to be inadequate for bone regeneration.^87^ As an alternative, silicate glasses are being increasingly considered for the treatment of bone infection. For instance, the silicate BG known as BoneAlive (S53P4) with a composition of (53SiO_2_–20CaO–4P_2_O_5_–23Na_2_O in wt %) was approved for clinical use in 2006 for the treatment of bone infections.^99,100^ BBGs may show an advantage for bone infection treatment over silicate glasses, as they convert more rapidly and completely to HA than silicate glasses; however, no commercial product based on BBGs for osteomyelitis treatment is available.^54^ BBGs can be loaded with antibacterial drugs such as gentamicin,^54,101^ teicoplanin,^87,102^ and vancomycin.^56,80,103^ In the study of Liu et al.,^76^ the release of vancomycin from Na_2_O–K_2_O–MgO–CaO–B_2_O_3_–P_2_O_5_ scaffolds increased rapidly initially, and after 3–4 days almost 100% of the drug was released from the BBG scaffolds. On the other hand, when cements were formed with the combination of chitosan, drug release was completed over 25 days. In this study, 87% of a rabbit tibia defect was recovered over 2 months.^104^ Xie et al.^80^ also used BBG (54B_2_O_3_–22CaO–Na_2_O–8K_2_O–8MgO–2P_2_O_5_ mol %) as a degradable local antibiotic delivery system for the treatment of chronic osteomyelitis. The BBGs were investigated as vancomycin carriers and delivery systems for eradication of osteomyelitis in rabbits. Bisphosphonate has also been loaded on a BBG carrier and results indicated that the drug-loaded BBG was efficient at inducing mineralization during in vitro and in vivo studies.^77^

Studies indicate that ion doping has been found to be efficient against bacteria.^38,94,95,98^ Silver oxide, tellurium oxide, cerium oxide, titanium oxide, zinc oxide, and gallium ions have been incorporated in various BBGs, and their antibacterial properties and cytocompatibility have been assessed. Adb-Allah et al.^95^ indicated that 2 mol % tellurium oxide doped 13-93B3 had higher antibacterial activity against *Staphylococcus aureus* (*S. aureus*) than 2 mol % zinc oxide, titanium oxide, and cerium oxide doped BBG. These BBGs showed low toxicity on human fibroblast cells. Mutlu et al.^98^ indicated that zinc oxide doping had a higher inhibitory effect against *S. aureus* (Gram-positive) bacteria than gallium doping. On the other hand, the two dopants had similar antibacterial effect against *Escherichia coli* (Gram-negative) bacteria. This effect was attributed to different thickness and cell wall structure of the two bacterial species, which made *Escherichia coli* more susceptible to damage from gallium ion doped 13-93B3 than *S. aureus*.

Singh et al.^105^ incorporated 30 vol % piezoelectric Na_0.5_K_0.5_NbO_3_ (NKB) and BaTiO_3_ phases in 1393B3 BBG powder to improve antibacterial properties and cellular response. The antibacterial activity increased by approximately 53% and 54% against *S. aureus* bacteria for BaTiO_3_ and NKB incorporated 13-93B3 glasses. This was explained as being due to electrostatic repulsion between BBG and the negatively charged bacterial membrane. The growth rate of MG-63 osteoblast cells was also enhanced after treatment with negatively polarized BaTiO_3_ and NKB incorporated BBG. Negatively charged surfaces enhanced the adhesion and proliferation of the osteoblast cells.

BBGs have been incorporated also in PMMA,^10,69,70^ chitosan,^26^ polycaprolactone (PCL),^34,64^ gelatin,^63^ and PVA forming bioactive composites.^66^ This strategy led to production of bone scaffolds with improved mechanical properties. Table 2 shows the list of BBG-incorporated polymeric matrices that have been developed for bone tissue healing.

**Table 2:** Studies of BBGs (All Melt-Derived) Incorporated Polymeric Matrices for Bone Healing

composition	findings
20, 30, 40% 13-93B3 in PMMA cement	5, 33, 100 μm BBG successfully added in PMMA^69^
20, 30, 40% 13-93B3 in PMMA cement	Modulus and compressive strength of 3 GPa and 130 MPa, respectively were achieved^10^
10, 20, 30% SrBG in PMMA cement	Modulus and compressive strength of 3.15 GPa and 90 MPa, respectively were achieved. % viability of MC3T3-E1 cells after treatment with cements showed biocompatibility of the composite^70^
13-93B3 in chitosan-based scaffold	Injectable scaffolds were successfully prepared.
	Compressive strength of up to 30 MPa was obtained.
	Up to 50% of the scaffolds degraded in 30 days^26^
13-93B3 scaffold with PCL coating	Compressive strength of 240 MPa was achieved^64^
13-93B particles coated with WS_2_ incorporated PCL/PLGA	0.1–2 wt % WS_2_ particles improved strength and in vitro bioactivity. Up to 1 wt % WS_2_ nanoparticles improved % MC3T3-E1 cell viability^65^
Particles coated with PCL/PLGA/hexagonal boron nitride	Compressive strength of 3.23 MPa was achieved after addition of 0.2 wt % boron nitride. Samples were found biocompatible with MC3T3-E1 cells^106^
13-93B3 in gelatin with citric acid scaffold	Highly bioactive injectable scaffolds were successfully achieved^63^
13-93B3 with platelet rich plasma scaffold	Incorporation of platelet rich plasma improved bone healing, in vivo^71^

### 4.2 Soft Tissue Engineering

BBGs are attracting increasing interest for soft tissue engineering applications.^33,56,58^ There is special interest in the exploitation of BBGs for chronic wound healing.^51,73^ Wound healing occurs in four stages including hemostasis, inflammation, cell proliferation (cell migration, angiogenesis, re-epithelialization, production of extracellular matrix), and maturation of the tissue.^107−109^ Angiogenesis enables transport of oxygen, nutrients, and growth factors which are critical for the wound healing process.^110^ A basic characteristic of nonhealing wounds is reduction of vessel formation around the wound area; therefore, promotion of angiogenesis is key for healing.^111,112^ It is very challenging to achieve angiogenesis in complex and thick tissues.^113^ As mentioned earlier, boron has been shown to stimulate angiogenesis.^14^ This effect has been related to stimulation of specific growth factors around the wound by the ionic dissolution products of BBGs.^66,112^

Boron takes part in the synthesis of extracellular matrix and stimulates secretion of collagen and proteins.^112^ Previous studies have shown that in a dose-dependent manner, boron can stimulate HUVEC proliferation and migration associated with the MAPK signal pathway.^31^ Moreover, boron promotes keratinocyte migration which also triggers the wound healing process.^108,114^ Wound healing is also promoted by up-regulation of the vascular endothelial growth factor (VEGF).^73^ It is also an antiseptic which aids the wound healing process.^51^ All of these studies indicate that boron has an important role in many different stages of wound healing.

**Figure 9:**
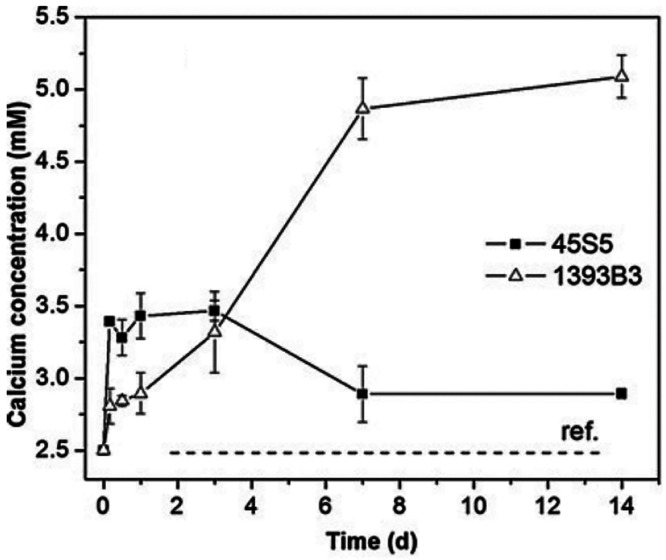
Calcium ion release rates from 13-93B3 and 45S5 BG microfibers in SBF showing significantly higher concentration of calcium concentration after 7 days from 13-93B3^9^ (Reproduced with permissions from ref (9). Copyright 2013 Springer).

Microfibrous 13-93B3 scaffolds exhibit rapid and full degradation, slow crystallization of ACP, and a higher concentration of dissolved calcium ions in SBF in comparison to 45S5 BG.^9^ In the final stages of the wound healing cascade, calcium ions are required in epidermal cell migration and regeneration, although the exact healing process has not been established. Importantly, the calcium ion concentration at the wound site should be compatible with events in the healing cascade.^9^Figure 9 shows release rates of calcium ions from 13-93B and 45S5 BG fibers.^9^

**Figure 10:**
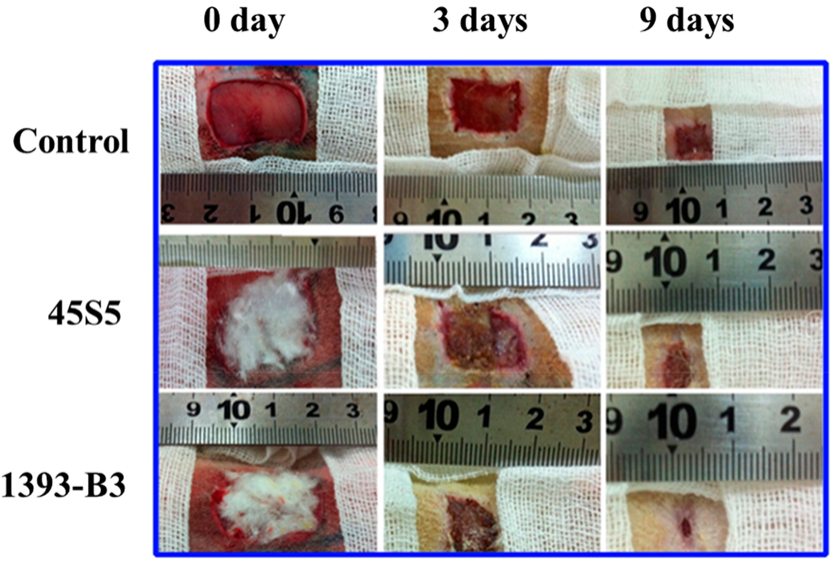
Skin wounds of Sprague–Dawley rats with no treatment (control) and groups treated with 45S5 BG and 13-93B3 microfiber wound dressings for 0, 3, and 9 days^115^ (Reproduced with permissions from ref (115). Copyright 2016 Elsevier).

As can be seen from Figure 9, calcium ion release rate is much higher for 13-93B3 than for 45S5 BG microfibers. This may partly explain higher wound healing capacity of 13-93B3 than silicate glasses.^9^ However, there are still questions about the exploitation of mineralizing glasses in soft tissue repair, e.g., the formation of HA layer on the BBG surface may not be required in wound healing.^27,61^ In contrary, some studies indicate that formation of an HA layer in the wound area triggers healing factors such as the antigen hematopoietic form precursor (CD44), the vascular endothelial growth factor (VEGF) precursor, and the vascular cell adhesion protein precursor, which lead to the assembly of epidermal cells at the site of injury. This eventually supports new tissue formation.^115,116,85^ Zhou et al.^115^ treated full-thickness dermal wounds on Sprague–Dawley rat skin with borate 13-93B3 and silicate 45S5 microfibers. In parallel with this, Lin et al.^117^ also implanted 13-93B3 and 4S5S BG microfibers in subcutaneous tissue of Sprague–Dawley rats, and a higher microvascular density was observed for 13-93B3 treated groups. As shown in Figure 10, wounds treated with 13-93B3 microfibers led to more rapid wound healing than 45S5 BG microfibers.^115^

For healing skin wounds, Mirragen a commercial product made of 13-93B3 glass microfibers has been approved by the U.S. Food and Drug Administration (FDA) in 2016.^10,47,48^ These microfibers have a cotton candy like structure imitating a fibrin clot microstructure.^118^ Human trials indicated that chronic wounds, such as diabetic foot ulcers and bedsores, healed in 6–10 weeks after application of Mirragen microfibers.^119−121^ This technology is described to be an effective treatment for wounds which exhibit no healing with conventional treatment options.^120,122^ Other advantages of these nanofibers are stated to be their easy handling and possibility to fit irregularly shaped wounds.^120^

BBGs were also incorporated in polymeric scaffolds for wound healing applications. For example, 13-93B3 with 5 mol % SrO particles in PVA hydrogel were produced. BBG acted as a filler and a cross-linking agent and improved mechanical properties. For these scaffolds, a compressive modulus of 0.12 MPa and an elastic modulus of 0.4 MPa were achieved. Boron ion release was less than 100 ppm which is lower than toxic levels.^66^ In another study, 10, 20, 40 wt % of 13-93B3 particles were added in methyl cellulose hydrogel. Methyl cellulose (MC) was cross-linked with manuka honey, which is a natural and biocompatible cross-linker of cellulose. It also has additional benefits such as antibacterial activity and wound healing capability. Samples were 3D printed with a nozzle size of 20G and a pressure of 550 kPa. The printing speeds were optimized depending on the BBG loading and the optimized printing speeds were 2 and 4.5 mm/s for MC and 40 wt % BBG/MC scaffolds, respectively. A compressive strength of 15 kPa was obtained for 40 wt % BBG incorporated samples with a pore size of 0.9 mm. This was found to be three times higher than that of the pristine MC hydrogel. Incorporation of BBG in the system improved printability of the scaffolds. In vitro studies with human dermal fibroblasts (hDFs) showed the biocompatibility of the 3D printed scaffolds for wound healing.^67^ Overall, incorporation of BBGs in polymers is observed to slow down boron ion release rate, prevent toxicity, and also improve mechanical properties. It is however remarkable that BBG containing inks for 3D bioprinting have not been extensively investigated to date.

**Figure 11:**
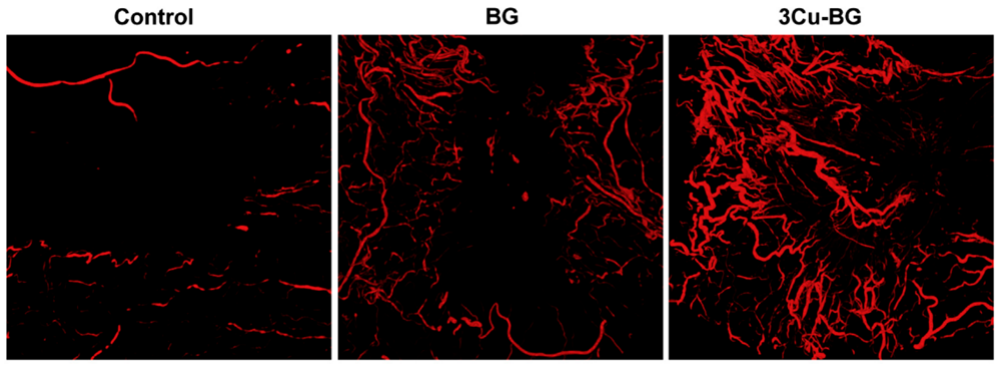
3D reconstructive images showing formation of blood vessels with no treatment (control), after application of 13-93B3(BG), and 3 wt % copper ions incorporated 13-93B3 (3Cu-BG) microfibers in full thickness skin defects in rodents 14 days after surgery^44^ (Reproduced with permissions from ref (44). Copyright 2015 Elsevier).

The research so far indicates that by incorporation of different dopants (biologically active ions), the wound repair capability of BBG scaffolds can be enhanced.^14,30,40,43,44^ However, dopant concentration is critical, as above certain concentrations, the dopants may lead to toxicity to the cells.^39,123^ First, addition of copper ions to BBGs has many advantages. The addition of copper ions could impair the crystallization of ACP to HA, which has been found to be advantageous for cell proliferation.^29^ Copper ions have been shown to stimulate the proliferation of endothelial cells during in vitro culture.^12^ Studies indicate that copper ions induce a hypoxia mimicking condition, which leads to the upregulation of the expression of VEGF, which is the growth factor playing a critical role in the formation of blood vessels.^14^ Therefore, it is indicated that copper ions stimulate angiogenesis, as mentioned above.^12^ Zhao et al.^44^ applied up to 3 wt % copper ion doped 13-93B3 microfiber wound dressings in full thickness skin defects in rodents. After 14 days, the healed skin samples were analyzed by computed tomography after staining with Microfil. The results indicated the drastic increase of vessel formation with the incorporation of copper ions in 13-93B3 scaffolds.^44^Figure 11 shows 3D reconstructive images indicating blood vessel formation after application of the wound dressings.^44^

Antimicrobial properties of scaffolds have been reported after doping BBGs with copper, zinc, gallium, and silver ions.^14,39,89,124^Table 3 shows an overview of BBG scaffolds which have been doped with various biologically active ions for soft tissue engineering applications.

**Table 3:** Incorporation of Different Ions in Melt Derived BBG Scaffolds for Soft Tissue Engineering Applications

composition	dopant	findings
60B_2_O_3_–36CaO–(4 – *x*)P_2_O_5_–*x*Ag_2_O (in mol %) powder	0, 0.3, 0.5, 1 mol % silver ions	Silver doped glass inhibited bacterial growth while undoped glass did not show such effect. All groups were nontoxic to fibroblasts and kerotinocytes. 0.3 and 0.5 mol % silver ion doped group reduced wound area^39^
13-93B3 fibers	0.4% copper oxide and 1% zinc oxide ions	Human skin fibroblast cells had high cell viability, growth, and migration ability^30^
13-93B3 powder	1% zinc, 3% copper oxide ions	Dendritic cell viability decreased with increase of copper oxide concentration to 3% and zinc ion concentration to 10%. Zinc and copper oxide ions avoid bacterial growth^14^
13-93B3 fiber	0.5, 1.0, and 3.0 wt % copper oxide ions	% cell viability of HUVEC and fibroblast cells increased up to 3% copper oxide ions over 7 days. Copper oxide ions enhanced wound repair capability^44^
13-93B3 scaffold	1, 3, 5 wt % cerium ions, 1 and 3 wt % vanadium, 1 and 5 wt % gallium ions	Cerium ions enhance angiogenesis while vanadium and gallium ions showed no such effect^43^
(52 – *x*)B_2_O–-16ZnO–14Na_2_O–CaO–P_2_O_5_–*x*Ga_2_O_3_ (in wt %) powder	2.5, 5, 10, and 15 wt % gallium ions	Gallium ions increased antibacterial effect^40^
13-93B3 powder	Cobalt, iron, gallium, iodine, strontium, and zinc ions	Priming with ion doped BBG increased the homing capacity of adipose stem cells^79^

Earlier, Poon et al.^123^ indicated that silver ions may also harm fibroblast and keratinocyte cells while killing bacteria. Naseri et al.^39^ studied the effect of silver ion doped BBG (60B_2_O_3_–36CaO–(4 – *x*)P_2_O_5_–*x*Ag_2_O), on *P. aeruginosa* bacteria as well as fibroblasts and kerotinocytes. The results indicated a dose-dependent reduction of bacteria after the silver doped BBG treatment. On day 4 of cell culture experiments, 0.375 and 0.75 mg/mL of BBG treatment with 0.5 mol % of silver ions, % keratinocyte cell viability increased, whereas 1.5 mg/mL of BBG treatment led to a decline of % cell viability. The study also indicated that 0.3 and 0.5 mol % doping of BBG led to kerotinocyte migration and promoted wound healing. Gallium and zinc ions increase immune tolerance both in vitro and in vivo.^14,40,98^ Deliormanlı et al.^43^ implanted porous BBG scaffolds in the connective tissue of the subcutaneous area of Sprague–Dawley rats, and the histological study indicated that incorporation of up to 5 wt % of cerium oxide ions into the 13-93B3 network significantly increased blood vessel formation. Despite its antibacterial properties, incorporation of up to 3 wt % of gallium ions into 13-93B3 scaffold was shown to reduce angiogenesis in a rat subcutaneous implant model. In the same study, doping 13-93B3 with 3 wt % vanadium ions was also proven to reduce angiogenesis.

In a few studies, nerve regeneration capabilities of BBGs were also studied. Marquardt et al.^33^ incorporated 13-93B3 in aligned fibrin microfibers and examined the viability of embryonic chick dorsal root ganglia (DRG). The study indicated that the % cell viability increased with the incorporation of BBG. Additionally, neural extensions were observed which indicated the potential use of BBG for neural tissue engineering applications. Gupta et al.^68^ incorporated 50 wt % 13-93B3 in PCL fibers for neural regeneration. In the study, different dopants were also incorporated in 13-93B3 to determine their effect on DRG outgrowth. The results indicated that 0.4 wt % iron, 1 wt % gallium, and 5 wt % zinc ion incorporated 13-93B3/PCL fibers led to significant neurite outgrowth. Another promising application of BBG is in muscle regeneration. Jia et al.^125^ studied the effect of 13-93B3 on muscle healing. 13-93B3 extracts were observed to stimulate secretion of CX43 and IG-1 from C2C12 cells. Also, in vivo studies were carried out with Sprague–Dawley rats with 7 mm of tibialis anterior muscle defects. Examination of the defect region under confocal laser scanning microscopy after BBG treatment led to improved vascularization compared with 45S5 BG powder. In the literature, cartilage tissue engineering is also suggested as a potential application of BBGs; however, to the authors knowledge, so far there are no research outcomes reported in this field.^82,86^

## 5 Conclusion

In several applications, BBGs are advantageous over silicate glasses due to a faster conversion rate to amorphous CaP. On the other hand, despite fast conversion to CaP, BBG converts to HA more slowly than silicate glasses, indicating their suitability for soft tissue repair. This slow conversion also increases the in vitro cell viability. BBGs of different compositions have been found to stimulate angiogenesis and osteogenesis. These effects are enhanced by doping BBGs with different ions. The following ions have been investigated in BBGs: copper, magnesium, strontium, cerium, silver, gallium, tellurium, vanadium, cobalt, iron, titanium, and iodine, with studies leading to different outcomes.

Most BBGs investigated so far have been produced by the melt-quenching route; however, production of BBGs via sol–gel processing may be preferable, as this leads to a greater surface area and porosity which ultimately increases bioactivity. However, analysis of the literature indicates that to date the sol–gel route has seldomly been applied for the preparation of BBGs. Therefore, greater research efforts are required to manufacture sol–gel processed BBGs with ion doping. Research so far also lacks sufficient work on 3D printing to prepare BBG scaffolds, which needs to be exploited further for preparation of patient-specifically designed scaffolds, especially scaffolds with sufficient mechanical properties for bone tissue engineering. In this context, biopolymer/BBG composite scaffolds have also received limited attention, even if they promise to be an effective approach to expand the applications of BBGs in soft tissue repair, applying techniques such as electrospinning and exploiting the angiogenesis porperties of BBGs. Moreover, although BBG scaffolds show promise for nerve regeneration, the field is in its infancy. Other promising research fields for BBGs are muscle and cartilage tissue engineering. Therefore, more research efforts are required to explore these potential application fields for new compositions of BBGs. Research should further focus on composites by smart combinations of biopolymers and BBGs. We expect that this review has provided a state-of-the art overview of the field of BBGs, and will prompt more studies regarding new compositions and applications of BBGs in tissue engineering and other biomedical applications.
